# Patient-reported outcomes after incisional hernia repair

**DOI:** 10.1007/s10029-021-02477-7

**Published:** 2021-08-02

**Authors:** N. van Veenendaal, M. M. Poelman, B. van den Heuvel, B. J. Dwars, W. H. Schreurs, J. H. M. B. Stoot, H. J. Bonjer

**Affiliations:** 1 Department of Surgery, Amsterdam University Medical Center, Boelelaan 1117, 1081 HV Amsterdam, The Netherlands; 2 Department of Surgery, Sint Franciscus Gasthuis, Rotterdam, The Netherlands; 3 Department of Surgery, Radboud University Medical Center, Nijmegen, The Netherlands; 4 Department of Surgery, Slotervaart Medical Center, Amsterdam, The Netherlands; 5 Department of Surgery, Noordwest Ziekenhuisgroep, Alkmaar, The Netherlands; 6 Department of Surgery, Zuyderland Medical Center, Sittard/Heerlen, The Netherlands

**Keywords:** Incisional hernia, Ventral hernia, Quality of life, Patient-centered outcomes, Patient-reported outcomes

## Abstract

**Purpose:**

Patient-reported outcomes (PROs) are pivotal to evaluate the efficacy of surgical management. Debate persists on the optimal surgical technique to repair incisional hernias. Assessment of PROs can guide the selection of the best management of patients with incisional hernias. The objective of this cohort study was to present the PROs after incisional hernia repair at long term follow-up.

**Methods:**

Patients with a history of incisional hernia repair were seen at the out-patient clinic to collect PROs. Patients were asked about the preoperative indication for repair and postoperative symptoms, such as pain, feelings of discomfort, and bulging of the abdominal wall. Additionally, degree of satisfaction was asked and Carolina Comfort Scales were completed.

**Results:**

Two hundred and ten patients after incisional hernia repair were included with a median follow-up of 3.2 years. The main indication for incisional hernia repair was the presence of a bulge (60%). Other main reasons for repair were pain (19%) or discomfort (5%). One hundred and thirty-two patients (63%) reported that the overall status of their abdominal wall had improved after the operation. Postoperative symptoms were reported by 133 patients (63%), such as feelings of discomfort, pain and bulging. Twenty percent of patients reported that the overall status of their abdominal wall was the same, and 17% reported a worse status, compared to before the operation. Ten percent of the patients would not opt for operation in hindsight.

**Conclusion:**

This study showed that a majority of the patients after incisional hernia repair still report pain or symptoms such as feelings of discomfort, pain, and bulging of the abdominal wall 3 years after surgery. Embedding patients’ expectations and PROs in the preoperative counseling discussion is needed to improve decision-making in incisional hernia surgery.

**Supplementary Information:**

The online version contains supplementary material available at 10.1007/s10029-021-02477-7.

## Introduction

Patients who develop incisional hernias have a reduced overall quality of life (QoL) because of the impact of incisional hernias on physical functioning and role functioning [1, 2]. Finally, 73–80% of patients require surgical repair [2, 3]. Reported success rates for incisional hernia repair vary considerably, depending on the primary outcome and the definition of a ‘successful’ incisional hernia repair. Whereas surgery-specific outcomes such as recurrence and complication rates are improving, a significant proportion of incisional hernia repairs are performed for symptom relief and to improve the QoL of the patient. The importance and hierarchy of surgical outcomes are subject to discussion [4].

In the last decade, the focus in hernia research has shifted from surgical outcomes, such as recurrences and complications, to patient-centered outcomes. Currently, outcomes such as chronic pain and QoL are frequently used as primary outcomes [5–7]. Since the goal of elective surgical interventions is to improve health-related QoL, measuring these patient-centered outcomes is of utmost importance [8].

Several instruments are used to measure QoL after incisional hernia repair [9]. Besides, an increasing number of organizations are collecting and assessing patient-reported outcomes (PROs). The concept of PROs serves to evaluate patients' point of view on outcomes [10]. The success of incisional hernia repair can be determined by adhering to the patient’s PROs.

Currently, there is no standardization of PROs in incisional hernia surgery and methodological quality of QoL instruments is poor [11]. Despite the increased emphasis on PROs in incisional hernia surgery, the correlation of PROs with preoperative indications and expectations is lacking. We aimed to elucidate preoperative expectations and compare them with postoperative PROs in incisional hernia patients.

## Methods

Patients were recruited from the multicenter prospective cohort study: the PINCH-Phone [12]. In short, the objective of the PINCH-Phone study was to develop and evaluate a new screening method for recurrent incisional hernias. Participants were asked questions by phone and underwent a physical examination at the out-patient clinic approximately 4 weeks after the phone call.

For this study, participants were invited to the out-patient clinic, and were retrospectively asked about the indication for their incisional hernia repair and prospectively interviewed about the postoperative PROs. Approval for this study was obtained from the Medical Ethical Committee of all participating hospitals in the Netherlands: Medical Center Alkmaar, Slotervaart Medical Center, VU University Medical Center, Zuyderland Medical Center Sittard/Heerlen. The STROBE guidelines were used to report this study [13].

Medical records of patients who had an operation code for ‘incisional hernia repair’ between January 2012 and December 2015 were screened. Patients with primary and recurrent incisional hernias were included. Patients with a history of complex abdominal wall treatment, presence of an ostomy, insufficient understanding of the Dutch language, or a mental disorder were not included in this study.

The interview questions are listed in Table 1. The interview took place at the out-patient clinic and lasted approximately 15 min. All interviews were carried out by one researcher, who was not involved with patient care, and not responsible for the healthcare-related consequences of the outcomes.

**Table 1:** Questions of the patient-reported outcomes’ measurement

Indication for surgery	
1	What was the indication for your hernia repair?
Satisfaction with surgery	
2	Considering the overall status of your abdominal wall, at the site of your incisional hernia repair: is it better, the same or worse after the operation?
3	Knowing the result of your incisional hernia repair, would you undergo the same procedure for an incisional hernia?
Current status	
4	Do you have any symptoms at the site of your incisional hernia repair? If yes, what are your symptoms?
5	Have you noticed anything at the site of your incisional hernia repair? If yes, what have you noticed?
6	Have you noticed something at the site of your incisional hernia repair when coughing, sneezing, or squeezing?
7	Could you please stand up and put one hand flat at the site of your incisional hernia repair. Put the other hand to your mouth and blow. Do you feel anything at the site of your incisional hernia repair?

Specifics of the incisional hernia and details of the method of repair were obtained from patients’ files.

The primary outcome of this study was the PROs on satisfaction and current status after incisional hernia repair. Secondary outcomes were the indication for repair and QoL scores, according to the Carolina Comfort Scale (CCS). The CCS is a validated, disease-specific questionnaire for patients after incisional hernia repair [14]. The CCS contains 23 items measuring the severity of pain, mesh sensation, and mobility impairment caused by the mesh for eight different movements: lying down, bending over, sitting up, activities of daily living, coughing or taking a deep breath, walking, walking the stairs, and exercise. The answers are recorded on a 6-point Likert scale, with 0 being an absence of symptoms to 5 being experiencing disabling symptoms. The CCS score is derived by the sum of all 23 items, with the best possible score being 0, and the worst possible score being 115. Symptom aggregates can be calculated as overall pain, mesh sensation, and movement limitation by taking the sum of all scores among all activities for the corresponding symptom. The maximum scores of each QoL domain were compared and a patient was considered symptomatic if the score exceeded 1 [14, 15]. The CCS has been proven to be valuable in hernia research and has been validated for the Dutch language [16].

Finally, physical examination was performed to check all patients for recurrences of the incisional hernia. A recurrence was defined as: ‘any abdominal wall gap with or without a bulge in the area of a postoperative scar, palpable or perceptible by clinical examination or imaging’ [17, 18]. Physical examination was performed both in standing and supine position. In case of doubt, an ultrasound was made. Specifics of the incisional hernia and details of the method of repair were obtained from patients’ files.

### Statistical analysis

Patient demographics were reported for the total cohort. Additionally, a subanalysis was done to study any differences in baseline characteristics between patients that went for open or laparoscopic repair. Descriptive statistics were reported as means with corresponding standard deviations for continuous variables and percentages for categorical variables. Categorical variables were evaluated using Pearson’s chi-squared test and Fisher’s exact test when appropriate. Pearson correlation coefficients were utilized to estimate the correlation between different postoperative PROs. Correlations were considered ‘strong’ if the coefficient value lied between 0.50 and 1, were said to be ‘moderate’ if the value lied between 0.30 and 0.49; and were considered ‘weak’ correlations if the coefficient lied below 0.29. A *p* value of < 0.05 was considered statistically significant. SPSS software version 23.0 was used to conduct all statistical analyses.

## Results

Medical records of 779 patients were screened and 621 patients were eligible for the study. These patients were sent patient trial information, and of these, 240 (39%) patients returned the informed consent forms. Nineteen patients could not be reached by telephone. Due to logistical matters, 11 patients could not make it for a hospital visit. Finally, 210 patients visited the out-patient clinic and underwent an in-depth interview, between January 2016 and December 2016.

### Baseline

The study population consisted of 105 males (50%) and 105 females (50%). The median age at operation was 58.4 years (range 20–93) and BMI 29.8 kg/m^2^ (range 17.7–53.1). The mean interval between index hernia repair and enrollment in the study was 38 months (range 12–49). Ninty-nine patients (47.1%) had undergone laparoscopic incisional hernia repair, and 111 patients (52.9%) had undergone conventional open repair. In the open repair group, 94 patients (84.7%) underwent mesh repair and 17 patients (15.3%) non-mesh, suture repair (Table 2).

**Table 2:** Baseline characteristics

	Total	Open repair	Laparoscopic repair	*p* value
*N*	210	111	99	
Sex (female)	105 (50%)	52 (46.8%)	53 (53.3%)	0.33
Age at operation, mean (SD)	58 (13)	58 (14)	59 (11)	0.51
BMI in kg/m^2^, mean (SD)	29.8 (5.7)	28.7 (4.8)	31 (6.3)	0.002
Hernia frequency				
Primary incisional hernia repair	154 (73%)	86 (77%)	68 (69%)	
Recurrent incisional hernia repair	56 (27%)	25 (23%)	31 (31%)	0.15
Placement of mesh				
No mesh	18 (8.6%)	17 (15.3%)	1 (1.0%)	< 0.001
Mesh	192 (91.4%)	94 (84.7%)	98 (99%)	
Hernia classification				
W1 (< 4 cm)	78 (37.1%)	40 (36%)	38 (38.4%)	0.82
W2 (4–10 cm)	90 (42.9%)	47 (42.3%)	43 (43.4%)	
W3 (> 10 cm)	42 (20%)	24 (21.6%)	18 (18.2%)	
Interval from operation until visit to out-patient clinic, months (IQR)	38 (27, 45)	40 (29, 48)	35 (26, 42)	0.003

Respondent and non-respondent characteristics were compared and found to be similar in terms of age, sex, hernia type, and repair techniques. The non-responder group of 411 patients consisted of 44% men. The median age of the 411 non-responders was 59 years (range 24–93). Sixty-two percent underwent an open repair and 38% a laparoscopic repair.

### Indication for surgical repair

Two hundred and three patients (96.7%) could recall the indication for incisional hernia repair. One hundred and seventy-two patients mentioned at least two indications, 83 patients reported at least three indications, and 12 patients remembered four indications for surgical repair. Seven patients (3.3%) could not remember the reason for incisional hernia repair (Table 3). One hundred and seventy-six patients (84%) mentioned the presence of a bulge as surgical indication, of which 124 times (60%) as primary reason for repair. One hundred and forty-eight patients reported the feelings of discomfort as important indication for surgical repair. Pain was the third indication for repair mentioned by 85 patients, of which 40 times (19%) reported as primary indication. Other reasons for repair mentioned by patients were aesthetics (5%), episodes of incarceration (2%), recurrence (2%), advice of the surgeon (2%), and acute incarceration requiring direct surgical repair (1%).

**Table 3:** Indications for incisional hernia repair according to patients

	Primary raison for repair	Secondary reasons for repair	Total times mentioned
Presence of a bulge	124 (60%)	52 (19%)	176 (37%)
Pain	40 (19%)	45 (17%)	85 (18%)
Discomfort	11 (5%)	137 (51%)	148 (31%)
Episodes of incarceration	3 (1%)	7 (3%)	10 (2%)
Aesthetic	3 (1%)	20 (7%)	23 (5%)
Recurrence	7 (3%)	3 (1%)	10 (2%)
Acute incarceration	7 (3%)	–	7 (1%)
Advice of the surgeon	8 (4%)	3 (1%)	11 (2%)
Don’t know	7 (3%)	–	7 (1%)
	210 (100%)	267 (100%)	477 (100%)

### Satisfaction with surgery

In total, 132 patients (63%) thought that the overall status of their abdominal wall was better than before the operation. Seventy-eight patients (37%) reported that either the overall status of their abdominal wall was the same or worse than before the operation (Table 4). Additionally, 188 patients (90%) said that they would opt for an operation again. In total, 22 patients (10%) said that they would not opt for an operation again.

**Table 4:** Satisfaction after incisional hernia surgery

Question	Total (*n* = 210)
Overall status of the abdominal wall compared with the situation before the repair?	
Better	132 (63%)
Similar	42 (20%)
Worse	36 (17%)
Would you undergo an incisional hernia repair again?	
Yes	188 (90%)
No	22 (10%)

### Current status/PROs

One hundred and thirty-seven patients (65.2%) answered ‘yes’ to one or more questions of the PINCH-Phone [12]. Eighty-nine patients (42%) reported symptoms 3 years after incisional hernia repair (Table 5). Although 121 patients reported negatively to the question whether they had any symptoms, 44 patients did use the open field to specify the symptoms they had. In total, 133 patients (63%) reported 184 symptoms. Symptoms reported were feelings of discomfort (30%), mild but bothersome pain (21%), bulging (13%), severe pain/pain in rest (12%), pain during exercise only (7%), abdominal complaints (4%), and aesthetic complaints (1%) (Table 6). Seventy-seven patients (36.7%) had no symptoms.

**Table 5:** Reponses from participants on the current status of their abdominal wall

Question	Total (*n* = 210)
Do you have symptoms related to surgical site?	
Yes	89 (42%)
No, but	44 (21%)
No	77 (37%)

**Table 6:** Symptoms related to the site of the incisional hernia repair reported by patients

Symptoms*	Total (*n* = 210)
Presence of a bulge	27 (13%)
Severe pain/pain in rest	26 (12%)
Mild but bothersome pain	45 (21%)
Pain at exercise	13 (7%)
Feelings of discomfort	63 (30%)
Abdominal symptoms	8 (4%)
Aesthetic complaints	2 (1%)

*133 patients reporting 184 complaints

### Carolinas Comfort Scale

All 192 patients with a mesh completed the CCS at the out-patient clinic. The CCS was missing for 17 patients with open non-mesh repair and for one patient in the laparoscopic group. Mean CCS score was 9.48 (range 0–83), of which 52.6% (*p* = 0.32) were considered to be symptomatic. According to the CCS, 44% patients had ‘mild but bothersome’ to ‘disabling’ pain (Table 7).

### Physical examination

A clinical recurrence was found in 34 patients (16%): 14 in the open group and 20 in the laparoscopic group. Fifty-four patients (54%) that had underwent laparoscopic incisional hernia repair suffered from bulging.

**Table 7:** QoL score by CCS

Carolina Comfort Scale domain	Total (*n* = 192)
Mesh sensation	
Mean (SD)	2.3 (5.4)
Symptomatic patients*	44 (23%)
Pain	
Mean (SD)	4.4 (7.1)
Symptomatic patients	85 (44%)
Activity limitation	
Mean (SD)	2.8 (5.8)
Symptomatic patients	57 (30%)
Cumulative CCS score	
Mean (SD)	9.5 (15.5)
Symptomatic patients	101 (53%)

*Total scores exceeding 1 were considered symptomatic (ranging from ‘mild but bothersome’ to ‘disabling’﻿ symptoms)

### Correlations

Preoperative pain was moderately correlated with postoperative symptoms, *r* (48) = 0.310, *p* = < 0.001. The presence of a postoperative bulge was moderately correlated with satisfaction regarding the status of the abdominal wall, *r* (127) = 0.395, *p* = < 0.001. The different postoperative outcomes as reported by patients showed no correlation with the decision to undergo the same operation again. Severe pain as reported by patients was weakly correlated with higher scores on the CCS, *r* (22) = 0.319, *p* = < 0.001. Pearson correlation coefficients were weak for other various variables (range *r* = 0.01–0.26) (supplement).

## Discussion

This study showed that 63% of patients reported symptoms 3 years after incisional hernia repair. Symptoms reported were feelings of discomfort (30%), mild but bothersome pain (21%), bulging (13%), pain in rest (12%), pain at exercise (7%), abdominal complaints (4%), and aesthetic complaints (1%). According to the CCS, 44% patients had ‘mild but bothersome’ to ‘disabling’ pain, and 53% patients were symptomatic. Although a majority of patients was satisfied after the procedure, 37% experienced a similar or worse overall status of their abdominal wall compared with preoperatively.

Considering the reported indications for incisional hernia repair, the high percentage of persistent symptoms or pain is an unsatisfactory outcome. Patients with incisional hernias seek medical care for various reasons, such as bulging, pain, discomfort, limitation of daily activities, aesthetic complaints, skin problems, or incarceration with or without strangulation of the hernia content [19]. The presence of a bulge and feelings of discomfort were the most common indications for surgical repair in our study cohort. Discomfort is an important reason why patients seek medical care [13, 20, 21], and thus warrants symptom relief and improvement of QoL after surgery.

Our results contradict the conclusion of a questionnaire among hernia surgeons. The most important indications for repair according to the experts were pain and limitations in daily activities, whereas discomfort and aesthetic complaints were the least valid indications for repair [3]. The discrepancy between our findings and the outcomes of the questionnaire might be the result of the intent of the surgeon to restore an impairment of the abdominal wall and prevent further harm. In contrast, the concern of the patient is an uncomfortable, disfiguring lump. The discrepancy in indications for hernia repair according to patients and surgeons warrants alignment of indications.

As 53% of patients are considered to be symptomatic at 3 year follow-up, it is disappointing that a better outcome is not forthcoming. Pain was mentioned by 19% of patients as primary reason for surgical repair, but 44% of patients suffered from ‘mild but bothersome’ to ‘disabling’ pain at time of this study. Knowledge of the chance on postoperative pain should enhance shared decision-making at the outer patient clinic. Furthermore, as pain and discomfort are subjective terms, clear definitions can benefit the indication setting and postoperative evaluation.

The recurrence rate of 16% in our study cohort corresponds with incidences in other studies [2]. However, the outcome recurrence is an objective, surgical outcome that is of less interest to patients. There appears to be a mismatch in accepted outcomes between surgeons’ and patients’ perspectives. The relatively high percentage of bulging found at physical examination can be explained by the fact that brigded repairs without defect closure were performed. Since presence of a bulge was the primary indication for incisional hernia repair in 60% of the patients, laparoscopic repair seemed to have failed in achieving symptom relief in these patients. After all, patients expecting to get their bulge repaired, still experience the discomfort of a hernia in case of bulging.

The outcomes of this study show the relevance of evaluating PROs after incisional hernia repair, rendering the need of integrating patients’ expectations in the preoperative counseling discussion. In the last 2 decades, there has been an increased interest in QoL in surgical research [22–24]. Due to a decrease in recurrence rates in incisional hernia surgery [25, 26], the focus has shifted from objective, surgical outcomes to subjective, patient-centered outcomes [9]. In hernia research, PROs have gained importance in the evaluation of surgical outcomes [27, 28]. However, there are still important practical questions about how these outcomes should be collected, visualized, shared, and used to improve the quality of care [11]. Although, we could not identify strong correlations between preoperative indications and postoperative PROs, our results do show discussion points and input for future research to enhance shared decision-making in incisional hernia surgery.

Prior studies have quantified the outcomes after incisional hernia repair with questionnaires selected by surgeons. Hernia-specific tools that are used are the CCS, Activities Assessment Scale (AAS) and Hernia-Related Quality of Life Scale (HerQLes). Furthermore, various general QoL questionnaires are used with hernia-specific questionnaires, or as sole method. The heterogeneity in hernia tools is extensive, and standardization in hernia surgery is warranted [29]. However, before a core outcome set can be defined, questions should be raised on how to best define a successful repair and how to define the parameters for success. A balanced set of outcomes from the patient’s and the surgeon’s perspective should be developed.

Satisfaction with a surgical procedure is the relation between expectations and experience, and is a major determinant in defining successful repair [30]. The effect of a surgical intervention on QoL influences satisfaction after incisional hernia repair [31]. Although successful repair can be expected in the majority of cases, 15–32% of the patients after incisional hernia repair will get a recurrence or suffer from chronic pain. These unwanted outcomes are important determinants in the outcomes of incisional hernia repair with its reflection on satisfaction rates. Reasons for dissatisfaction after incisional hernia repair are impairment of physical function, scars, abdominal pain, and a disappointing aesthetic result [32]. Knowledge of expectations and satisfaction rates allows us to optimize shared decision-making in incisional hernia management.

Although our results show that 63% of the patients were satisfied, 37% reported that either the status of their abdomen was the same or worse compared to preoperatively. Our findings correspond with the previous studies showing satisfaction rates of 50%–86% at 5–10 year follow-up [33–36]. Furthermore, our findings reveal that 90% of the patients would opt for the operation again. With the knowledge of hindsight, many may wonder if patients would have opted for the operation as they seem to have been exposed to the risks of surgery with no clear benefit.

The CCS was used to quantify QoL after incisional hernia repair. Our results show that 53% patients can be considered symptomatic. This corresponds with 42% patients reporting symptoms related to the surgical site. The pain-domain of the CCS had the highest mean scores with 44% patients reporting postoperative pain. This resembles a study in which 38% of the mesh patients experienced occasional pain at the site of the operated area, 43% reported moderate immobility of the abdominal wall, and 31% experienced an unspecified foreign-body sensation [34]. Although the CCS is a disease-specific questionnaire, this questionnaire might not cover all quality-of-life-related complaints of patients.

Incisional hernia research is mostly featuring an ongoing debate regarding open versus laparoscopic surgical management looking at surgical outcomes [37]. Although laparoscopic repair is recommended compared with open repair when considering HRQoL [38], our results showed no difference in PROs between the two groups (data not shown).

This study has several limitations. First, participants were interviewed several years after incisional hernia repair and were asked about postoperative PROs only. Preoperative data were lacking, making a comparison impossible. The presence of prospective preoperative data would have strengthened our study design and is warranted in future research. The need for assessment of pre- and postoperative QoL differences using validated questionnaires has been acknowledged by other authors, as well [11]. With the current focus on PROs as primary outcome, more preoperative data are currently collected using validated questionnaires. It is also recommended to report the level of pre- and postoperative pain to determine the success of an incisional hernia repair. A second limitation is the lack of pre- and postoperative data on the use of analgesics in this study. It could have strengthened the study to have information on analgesics use, and including analgesic consumption in future studies is recommended. Our response rate of 39% can be considered a third limitation. Despite this relatively low response rate, it is still higher than the average 25–30% when no follow-up or reminder is sent [39]. Of the 621 patients that were approached, a potential bias can be that only patients participated with symptoms, that suffered from postoperative complications or that felt mistreated during their hospital stay. Patients that suffer from complaints are more likely to participate than those who do not have complaints at all [27]. Finally, a limitation of the correlation analysis is that only two variables are explored. Effects of other variables are not taken into account. Furthermore, estimation of the Pearson's correlation coefficient is sensitive to data distribution and is not robust. Outliers might have had their influence on the statistic *r*. Interpretation of the strength of the associations should be considered in light of this lack of robustness.

## Conclusion

Sixty-three percent of patients reported symptoms 3 years after incisional hernia repair, mostly feelings of discomfort, pain, and bulging. Thirty-seven percent of patients experienced a similar or worse status of their abdominal wall compared to preoperatively. Considering the main indications for incisional hernia repair to be presence of a lump and discomfort, the high percentage of symptoms and pain is an unsatisfactory outcome for elective incisional hernia repair. The outcomes of this study show the relevance of evaluating patient-reported outcomes after incisional hernia repair, rendering the need of integrating patients’ expectations in the preoperative counseling discussion.

## Supplementary Information

Below is the link to the electronic supplementary material.

Supplementary file1 (DOCX 196 KB)

Supplementary file2 (DOCX 346 KB)

Supplementary file3 (DOCX 290 KB)

Supplementary file4 (DOCX 14 KB)
