# Supplementary figures and images for: Patient-reported outcomes after incisional hernia repair
Source: Hernia. 2021 Aug 2;25(6):1677–84. doi: 10.1007/s10029-021-02477-7 (PMC8613099; doi:10.1007/s10029-021-02477-7)

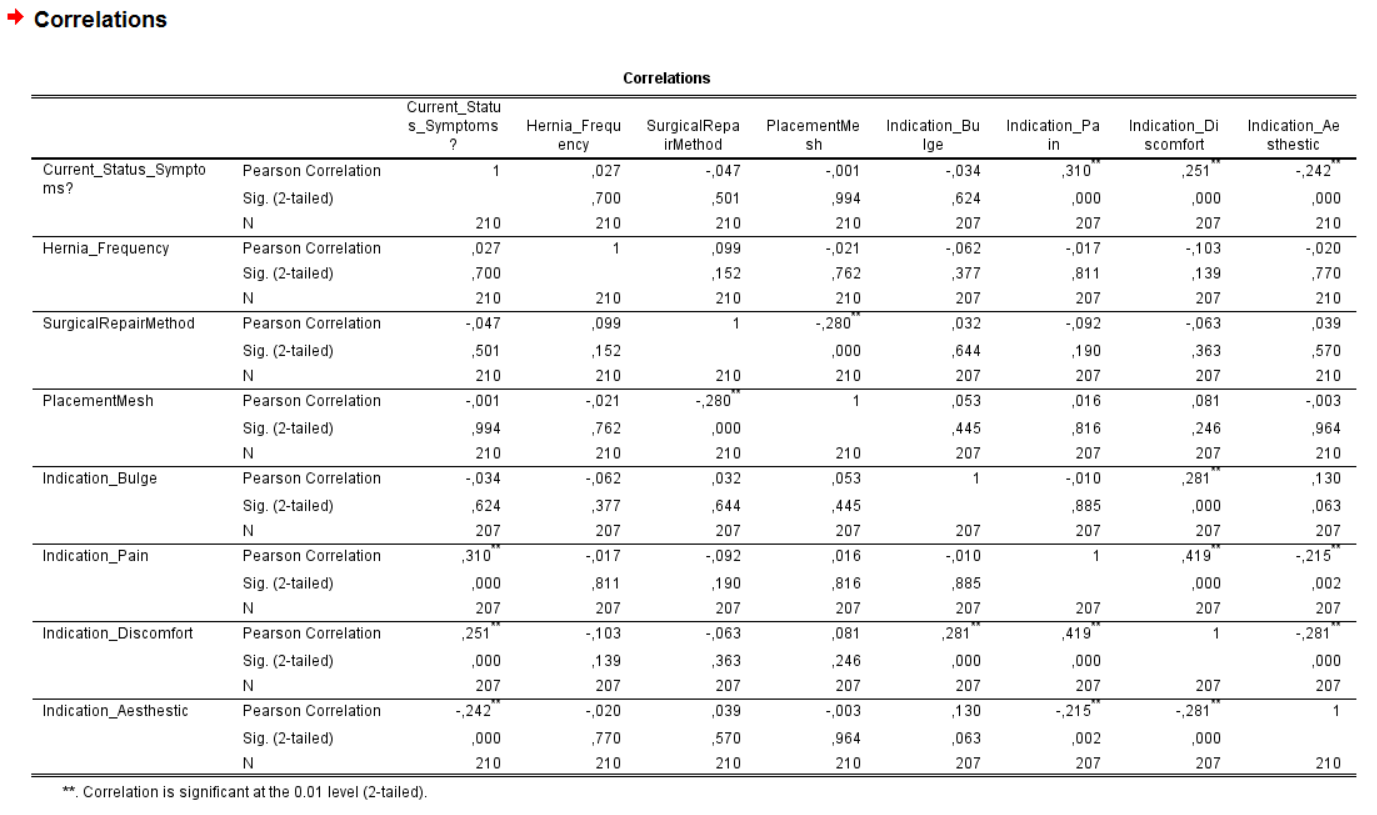

Supplement: Supplementary file 1 — Supplementary file1 (DOCX 196 KB) [file 10029_2021_2477_MOESM1_ESM.docx]

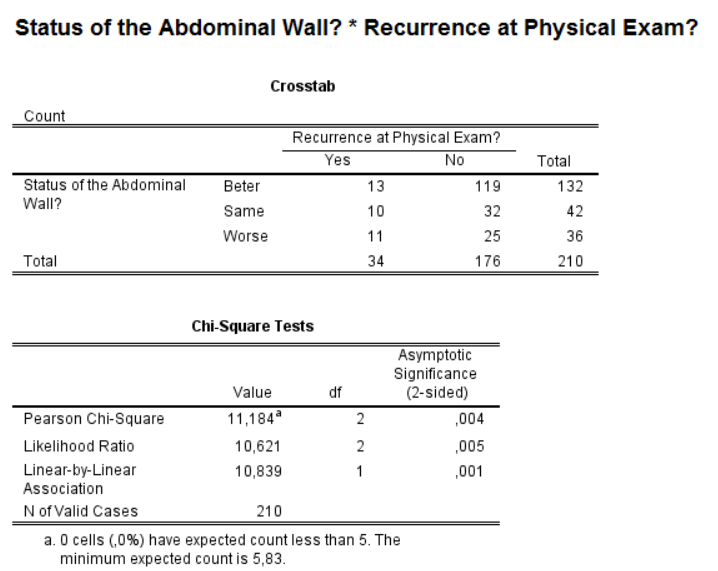


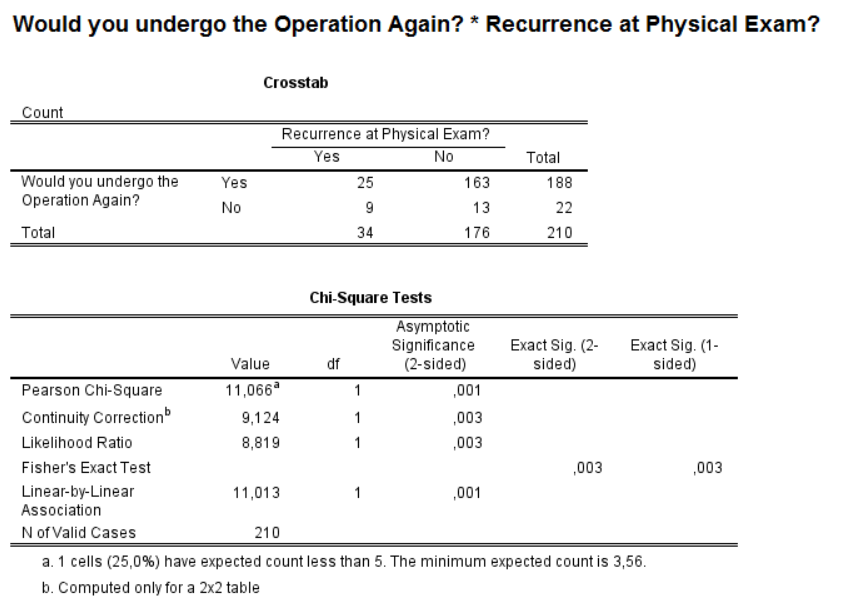


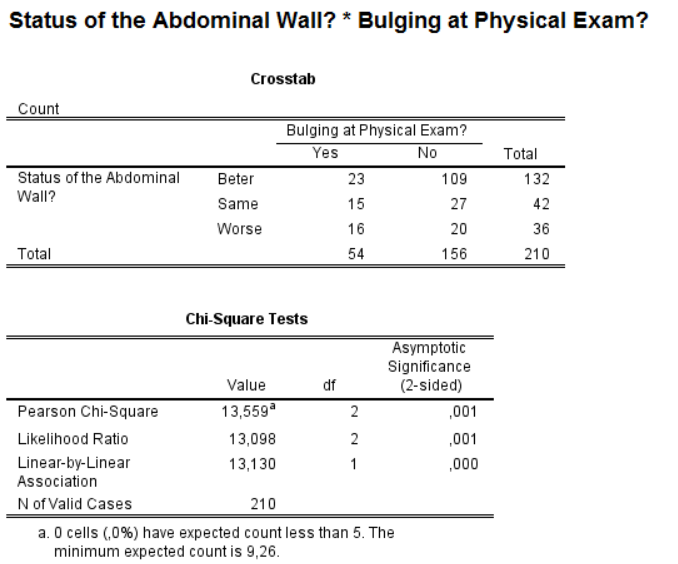


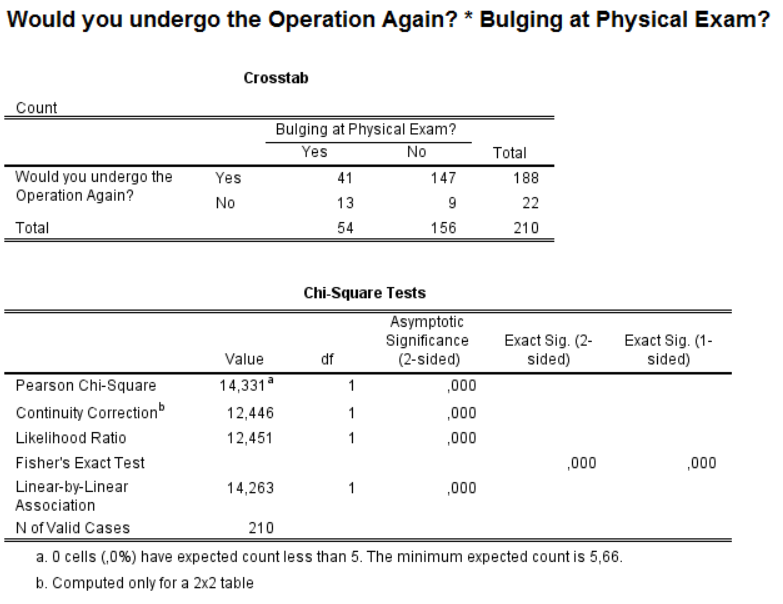

Supplement: Supplementary file 2 — Supplementary file2 (DOCX 346 KB) [file 10029_2021_2477_MOESM2_ESM.docx]

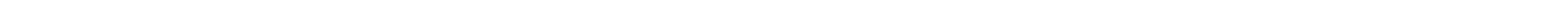

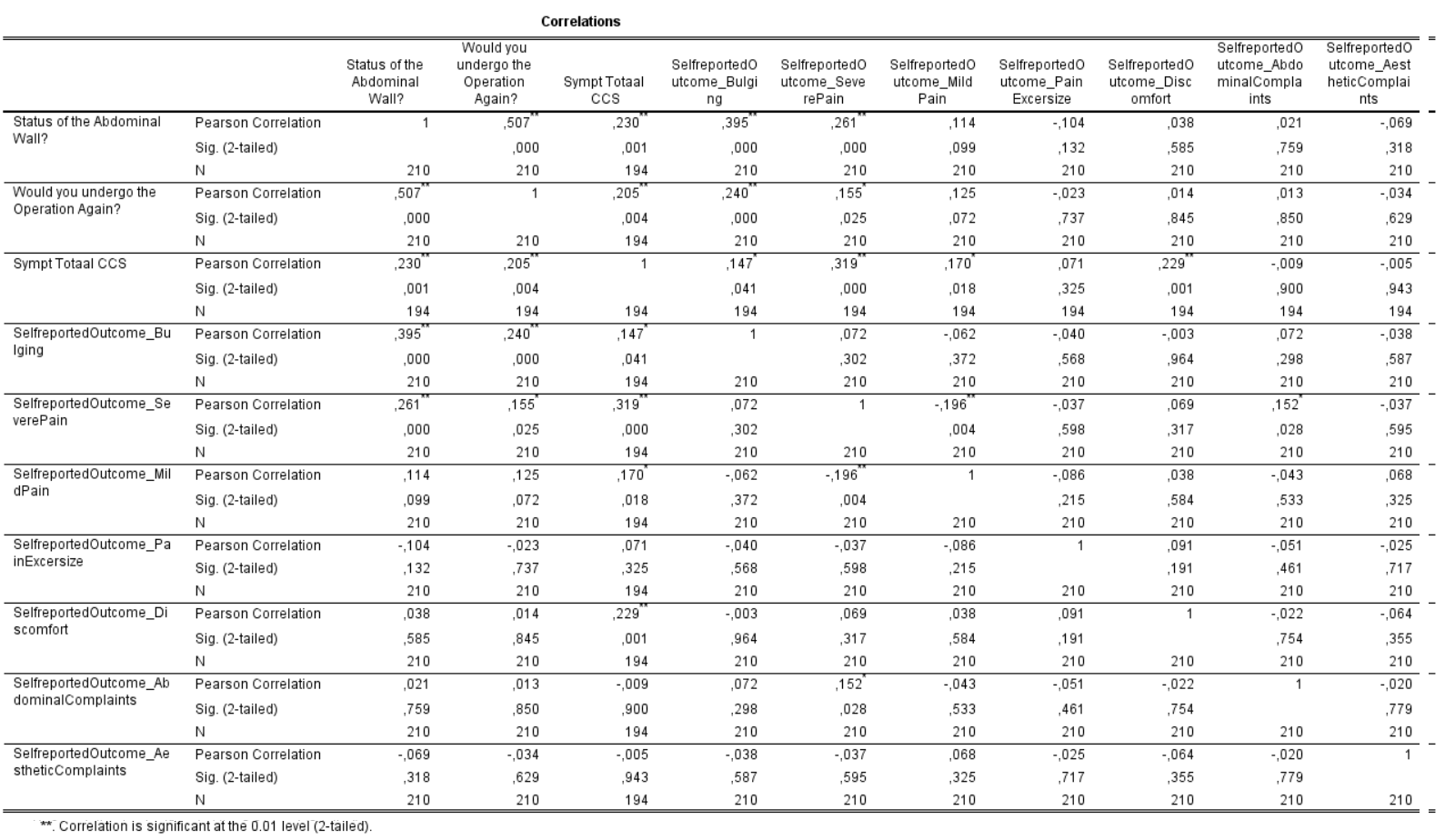

Supplement: Supplementary file 3 — Supplementary file3 (DOCX 290 KB) [file 10029_2021_2477_MOESM3_ESM.docx]
