# Supplementary material for: Patient-reported outcomes after incisional hernia repair
Source: Hernia. 2021 Aug 2;25(6):1677–84. doi: 10.1007/s10029-021-02477-7 (PMC8613099; doi:10.1007/s10029-021-02477-7)
Supplement: Supplementary file 4 — Supplementary file4 (DOCX 14 KB) [file 10029_2021_2477_MOESM4_ESM.docx]

**SUPPLEMENT**

| **Table 8. Correlation between preoperative symptoms and postoperative symptoms** | | |
| --- | --- | --- |
|  | Current status: symptoms? | |
|  | Pearson correlation coefficient | *P*-value |
| **Preoperative variable** |  | |
| Hernia frequency | 0.03 | 0.70 |
| Surgical repair method | -0.05 | 0.50 |
| Placement of a mesh | -0.05 | 0.05 |
| **Preoperative indication** |  | |
| Bulge | -0.03 | 0.62 |
| Pain | 0.31 | <0.001** |
| Discomfort | 0.25 | <0.001** |
| Aesthetic | -0.24 | <0.001** |

*correlation is significant at the 0.05 level (2-tailed)

** correlation is significant at the 0.01 level (2-tailed)

| **Table 9. Satisfaction after incisional hernia surgery for patients with recurrences or bulging at physical examination** | | | | | |
| --- | --- | --- | --- | --- | --- |
| Question: |  | Recurrences  (n=34) | *P*-value | Postoperative bulging (n=54) | *P*-value |
| **Overall status of the abdominal wall compared with the situation before the repair:** | Better  Similar  Worse | 13 (38%)  10 (29%)  11 (32%) | 0.004 | 23 (43%)  15 (28%)  16 (30%) | 0.001 |
| **Would you undergo incisional hernia repair again?** | Yes  No | 25 (74%)  9 (26%) | 0.001 | 41 (76%)  13 (24%) | <0.001 |

| **Table 10. Correlation of selfreported postoperative symptoms with satisfaction and CCS** | | | | | | |
| --- | --- | --- | --- | --- | --- | --- |
|  | Satisfaction regarding the status of the abdominal wall | | Satisfaction: Would you undergo incisional hernia repair again? | | Symptomatic patients according to Carolina Comfort Scale | |
| **Selfreported postoperative outcome** | Pearson correlation coefficient | *P*-value | Pearson correlation coefficient | *P*-value | Pearson correlation coefficient | *P*-value |
| Presence of a bulge | 0.40 | <0.001** | 0.24 | <0.001** | 0.15 | 0.04* |
| Severe pain/Pain in rest | 0.26 | <0.001** | 0.16 | 0.03* | 0.32 | <0.001** |
| Mild but bothersome pain | 0.11 | 0.10 | 0.13 | 0.07 | 0.17 | 0.02* |
| Pain at exercise | -0.10 | 0.13 | -0.02 | 0.74 | 0.07 | 0.33 |
| Feelings of discomfort | 0.04 | 0.59 | 0.01 | 0.85 | 0.23 | 0.23 |
| Abdominal symptoms | 0.02 | 0.76 | 0.01 | 0.85 | -0.01 | 0.90 |
| Aesthetic complaints | -0.07 | 0.32 | -0.03 | 0.63 | -0.01 | 0.94 |

*correlation is significant at the 0.05 level (2-tailed)

** correlation is significant at the 0.01 level (2-tailed)
